# Solving the puzzle of climacteric fruit ripening: EMB1444-like and its regulatory function

**DOI:** 10.1093/jxb/erad378

**Published:** 2023-11-21

**Authors:** Francesca Bellinazzo

**Affiliations:** Laboratory of Molecular Biology, Wageningen University and Research, 6708 PB, Wageningen, The Netherlands; Bioscience, Wageningen Plant Research, Wageningen University and Research, 6708 PB, Wageningen, The Netherlands

**Keywords:** Ethylene, climacteric fruit ripening, gene regulatory network, bHLH, transcription factor, transcriptional regulation

## Abstract

This article comments on:

**Zhao W, Wang S, Li W, Shan X, Naeem M, Zhang L, Zhao L**. 2023. The transcription factor EMB1444-like affects tomato fruit ripening by regulating *YELLOW-FRUITED TOMATO 1*, a core component of ethylene signaling transduction. Journal of Experimental Botany **74**, 6563–6574.

**Red, orange, yellow, green, striped: many colourful and tasty tomatoes are currently on the market all over the world. These fascinating varieties are obtained by targeting specific units of a complex gene regulatory network (GRN) of transcription factors, which work in concert to regulate the downstream effectors of tomato fruit ripening. Through a multi-angle approach,** **Zhao *et al*. (2023)** **unravel the regulatory role of the bHLH transcription factor EMB1444-like in the process of tomato fruit ripening, expanding the potential for developing elite tomato varieties that combine high-quality organoleptic and shelf-life characteristics.**

The fruits of tomato (*Solanum lycopersicum*) are climacteric, meaning that their ripening occurs through a self-sustained process that relies heavily on the synthesis and perception of the gaseous phytohormone ethylene and is characterized by a high level of cellular respiration in the fruit (Fig. 1) (Payasi and Sanwal, 2010; Kou *et al.*, 2021). Once the ripening cascade has been initiated, it will complete its course without any contribution from the mother plant; this allows growers to harvest climacteric fruits at early ripening stages, which are easier to handle and transport, and can be stored for longer than ripe fruits. Tomato is considered the model species for the study of fleshy fruit development and climacteric ripening, and the current knowledge on tomato ripening is quite extensive (Barry and Giovannoni, 2007). Nevertheless, many pieces of this challenging puzzle are still missing. Tomato fruit ripening is a highly complex trait, involving multiple effector genes that are finely regulated by a large GRN of transcription factors that affect each other’s expression and, ultimately, the expression of downstream effector genes (Kou *et al.*, 2021). A complete understanding of this GRN is key to being able to finely control fruit ripening-related traits and, ultimately, obtain a variety of beautiful, tasty, and long-lasting tomato fruits.

**Fig. 1.**
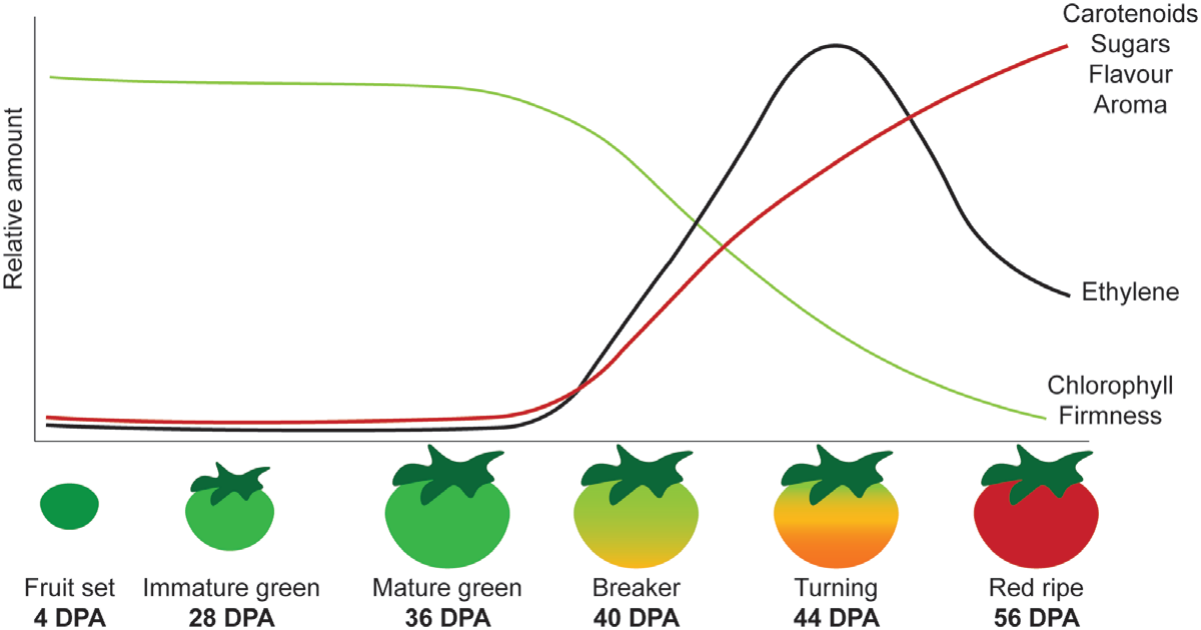
Schematic representation of tomato fruit ripening. At the early stages of ripening, tomato fruits are characterized by low ethylene content, high firmness, and relatively high chlorophyll content, which causes their green colour. At the same time, sugars and carotenoids, and therefore the perceivable aroma and flavour, are low. Starting from the breaker stage, at around 40 days post anthesis (DPA), a sharp increase in ethylene can be observed. Consequently, chlorophyll content and firmness gradually decrease, while carotenoids and sugars increase, together with aroma and flavour. As a result of these changes, the fruit colour changes towards yellow/orange or pink. Later on, at the turning stage (~44 DPA), ethylene levels decrease again, but the ripening cascade sustains itself until the fruits become deep red. Adapted from Wang *et al.* (2020a), with permission from Elsevier.

## EMB1444-like and the ethylene cascade

As in other climacteric fruits, the pivotal role of ethylene in tomato ripening has long been described. The ripening process begins with a flare of ethylene, which is synthesized in the fruit. Ethylene molecules bind to specific receptors and elicit the ethylene signalling cascade, which activates a final multifaceted response involving carotenoid accumulation, tissue softening, and the accumulation of sugars and flavonoids. The result: a juicy, red, tasty tomato.

One of the central molecular players in tomato ethylene signalling is the gene *SlEIN2*/*YELLOW-FRUITED TOMATO 1* (*YFT1*), which encodes a homolog of the Arabidopsis EIN2, which stabilizes EIN3 and other EIN3-like proteins to sustain the ethylene cascade. In this issue, Zhao *et al*. (2023) describe the bHLH transcription factor EMB1444-like as an upstream positive regulator of *SlEIN2/YFT1*.

EMB1444-like was selected as an important regulator of fruit ripening because of its expression profile and considering the results of an exploratory luciferase assay, which revealed that the presence of EMB1444-like was able to increase the expression of luciferase fused to the *YFT1* promoter. As a further step, an elegant experimental design based on yeast one-hybrid analysis and confirmed by electromobility shift assay was used to screen the promoter of *YFT1* to pinpoint the exact motif to which EMB1444-like can bind. This motif turned out to be an E-box (CACTTG), which is known to be one of the preferred DNA sequences for bHLH binding. To verify whether EMB1444-like affects the expression of *YFT1 in planta,* the latter was measured by qPCR in *EMB1444-like* knockdown lines (named *sledl* lines). As expected, *sledl* lines showed differential expression of *YFT1*, which appeared to be down-regulated relative to wild-type tomato fruits. By contrast, the expression of *EMB1444-like* was unchanged in *yft1* mutant lines. As a further piece of evidence supporting the hypothesis that EMB1444-like affects the downstream ethylene cascade, *sledl* lines showed decreased expression of ethylene biosynthesis genes such as *ACC SYNTHASE 2/4* (*ASC2/4*) and *ACC OXIDASE 1* (*ACO1*), and ethylene signalling genes including *APETALA 2a* (*AP2a*), *ETHYLENE-INSENSITIVE3-like 3* (*EIL3*) and *NEVER RIPE* (*NR*). As a consequence, the fruits of *sledl* lines emitted less ethylene than wild-type fruits, confirming the role of EMB1444-like as a positive regulator of the ethylene cascade.

In a previous study, Zhao *et al*. (2021) characterized another upstream regulator of *YFT1*, the transcription factor WRKY32. EMB1444-like and WRKY32, both of which are positive regulators of *YFT1*, and consequently of tomato ripening, have an additive effect. The expression of *EMB1444-like* and *WRKY32* is not affected in *yft1* lines, which produce less ethylene, and therefore their expression appears to be unaffected by any ethylene-mediated feedback loop. This observation could be explained by considering EMB1444-like and WRKY32 as upstream regulators of the ethylene cascade. In fact, *sledl* lines, as well as *WRKY32* knockdown lines (named *slwr*), produce less ethylene compared with their wild-type counterparts, suggesting that the ethylene cascade operates downstream of these transcription factors. These results differ from what has been observed for the MADS-box transcription factors SlFUL1 and SlFUL2. In that case, although displaying ripening phenotypes, *FUL1* and *FUL2* RNAi lines maintain an unaltered ethylene signalling cascade and ethylene production, and these transcription factors have been therefore identified as ethylene-independent regulators of fruit ripening (Bemer *et al.*, 2012).

## The effect of EMB1444-like on carotenoid biosynthesis and fruit ripening

The downstream effect of EMB1444-like on fruit ripening is evident: *sledl* lines show delayed maturation of chromoplasts, the specialized plastids that develop from chloroplasts in ripening fruits, which accumulate high levels of carotenoid pigments (e.g. lycopene, lutein, and beta-carotene) and confer the red colour of ripening tomato fruits. Accordingly, when *EMB1444-like* is silenced, the expression of carotenoid biosynthesis genes decreases. As a result, the fruits of *sledl* lines are green even 54 days after flower anthesis, a stage at which wild-type fruits are deep red and ripe.

Although the identification of new genes of the tomato fruit-ripening GRN is *per se* a stepping stone towards building a complete picture of it, the phenotypes of fruit-ripening mutants can sometimes be misleading, and therefore any evaluation of the functional role of their causal transcription factors needs careful consideration. Spontaneous tomato mutants with a similar stunted fruit-ripening phenotype to those observed in *sledl* and *slwr* lines have served as the basis for the molecular study of tomato fruit ripening. Among these, *ripening inhibitor* (*rin*) and *non-ripening* (*nor*) mutants showed severe ripening alterations, and therefore the causal mutated transcription factors (a MADS-box and a NAC-domain, respectively) have been long considered master regulators of fruit ripening (Karlova *et al.*, 2014). Recently, mutants for RIN and NOR have been obtained by gene editing using the current CRISPR-based technology, and the results were startling: the phenotypes of null mutant lines were much milder than those of the spontaneous mutants and knockdown lines obtained with RNAi (Wang *et al.*, 2019; Gao *et al.*, 2020). This phenomenon was explained by the fact that the *rin* and *nor* lines, among others, are characterized by dominant negative mutations, which block the function of other transcription factors as well, usually when protein complexes are formed. Moreover, RNAi can trigger non-specific silencing, increasing the phenotype severity. On the other hand, the adoption of frameshift mutations obtained by using CRISPR-Cas can lead to masked phenotypes because of compensatory mechanisms that increase the transcription of other, partially redundant transcripts (Wang *et al.*, 2020a). Ideally, CRISPR technology should be used to obtain tailored mutations where either the entire genomic locus is deleted or expression is completely suppressed, and therefore the production of aberrant transcripts that can elicit those compensatory responses is avoided (Wang *et al.*, 2020a).

## Knowledge applications: GRN fine-tuning

As is often the case, things are more complicated than they appear. Recent studies have contributed to unravelling the complexity of the tomato fruit-ripening GRN, a robust network of numerous transcription factors with partially redundant functions (Wang *et al.*, 2020a,b), which interacts with other aspects of the overall regulome (epigenetic factors, hormones, metabolites, etc.). This model is distinctly different from the original one, which contained a handful of master regulators. As the overall picture becomes increasingly sophisticated, the opportunities for combining ideal traits in a controlled and fine-tuned manner also multiply. Therefore, breeding for elite tomato varieties that combine mutations regulating the expression of target regulatory genes becomes possible. The results may soon be available in our kitchens.
